# Residual metal soil contamination and associated health risks in a native American community within a remediated Superfund site in New Jersey, USA

**DOI:** 10.1007/s11356-026-38049-4

**Published:** 2026-07-22

**Authors:** Tri Huynh, Eliane El Hayek, Abdulmehdi Ali, Tessa Hernandez, Jania Zenon, Connie Van Dunk, Vincent Mann, Esther Erdei, Judith Zelikoff

**Affiliations:** 1 Division of Environmental Medicine, Department of Medicine, New York University Grossman School of Medicine, New York, NY 10010 USA; 2 Department of Pharmaceutical Sciences, College of Pharmacy, Health Sciences Center, University of New Mexico, Albuquerque, NM 87131 USA; 3 Department of Earth and Planetary Sciences, University of New Mexico, Albuquerque, NM 87131 USA; 4 The Ramapough Lunaape Turtle Clan, Ringwood, NJ 07456 USA

**Keywords:** Environmental justice, Heavy metals, Human health risk assessment, Soil contamination, Source apportionment, Superfund

## Abstract

This study evaluated whether remediation at the Ringwood Mines/Landfill Superfund site in New Jersey reduced soil heavy metal exposure and associated health risks for the Ramapough Lunaape Turtle Clan, a Native American community whose traditional ties to the local land may increase exposure to legacy pollution. Soil heavy metal concentrations were measured in frequently visited areas in and around the Ringwood Superfund site, and evaluated against background levels using the geo-accumulation index (I_geo_) and ecological risk index (E_r_). I_geo_ results signaled anthropogenic enrichment of antimony, arsenic, cadmium, and lead, with cadmium showing E_r_ values denoting *very high* ecological risks. Receptor models (Positive Matrix Factorization [PMF] and UNMIX) identified source profiles consistent with iron mine tailings (arsenic, cobalt, iron, and vanadium) and automotive waste (cadmium, chromium, lead, and nickel), linking observed contamination to the sources responsible for the site’s Superfund designation. Monte Carlo simulations of the hazard index (HI) and total cancer risk (TCR) indicated potential non-cancer and cancer risks for children and adults, primarily through soil ingestion, dermal contact, and game meat consumption—an oft-overlooked pathway in conventional risk assessments. Despite claims that the site is under control for human exposure, soil contamination from legacy sources persisted at levels associated with excess health risks. Findings from this study highlighted potential deficiencies in current remediation strategies in protecting the health of Native American communities with cultural and subsistence ties to their land, and contributed evidence that can inform future studies in similar settings.

**Supplementary Information:**

The online version contains supplementary material available at 10.1007/s11356-026-38049-4.

## Introduction

The Ringwood Mines/Landfill Superfund site (hereinafter, referred to as the Ringwood Superfund site) (ID Number: NJD980529739) in northern New Jersey (NJ), represents a complex case of hazardous legacy contamination, regulatory shortcomings, and Indigenous marginalization. Located on the ancestral lands of the Ramapough Lunaape Turtle Clan (RLTC), the site has an industrial legacy beginning with iron mining operations from the mid-1700s through World War II (Hotz 1953). Following mine closure, the site became a repository for industrial and municipal waste disposal. Substantial quantities of paint sludge, solvents, and automotive manufacturing byproducts were deposited in abandoned mine shafts, buried underground, and dumped in open spaces across the site throughout the 1960 s and 1970 s (US Environmental Protection Agency n.d.). As a result, the site was first listed on the National Priorities List (NPL) as a Superfund site by the U.S. Environmental Protection Agency (EPA) in 1983, de-listed in 1994, and relisted in 2006, due to continued reports of exposure and adverse health outcomes; it remains the only Superfund site to have been relisted after deletion (US Environmental Protection Agency (US n.d.)).

As part of the Superfund remedial process, site investigations have documented elevated levels of heavy metals, such as antimony, arsenic, cadmium, and lead, among other toxic chemicals in soil and groundwater (New Jersey Department of Health and Senior Services 2006). Cadmium is linked to kidney damage and bone demineralization (Genchi et al. 2020), while chronic lead exposure may result in neurodevelopmental deficits in children and hypertension in adults (US Environmental Protection Agency 2024b). Arsenic is a carcinogen associated with skin, lung, and bladder cancers, as well as cardiovascular and neurological effects (Hong et al. 2014, International Agency for Research on Cancer 2012). Antimony, sharing analogous chemical properties with arsenic, elicits similar toxicological effects similar (Gebel 1997; Wysocki et al. 2023) and upon dermal contact, may cause distinctive rashes resembling chickenpox lesions, known as "antimony spots" (US Environmental Protection Agency 1980).

Metal exposure can occur via multiple pathways: soil ingestion and dermal contact, dust inhalation, or consumption of game harvested from polluted ecosystems (New Jersey Department of Health and Senior Services 2006). Many Native American communities, like the RLTC, choose to remain on their ancestral lands and continue traditional practices despite documented environmental contamination and associated health risks (Fernández‐Llamazares et al. 2020). For these communities, exposure and health risks are compounded by their reliance on the land for subsistence. Anthropological studies described hunting, foraging, and gardening (Mann et al. 2022) practices that increase the likelihood of exposure to soil-borne contaminants relative to the broader population. A health assessment study by Meltzer et al. (2020) revealed a significant association between being a Native American and Ringwood resident and self-declared opportunities for Superfund site exposure, which was in turn significantly associated with higher odds of bronchitis and asthma (Meltzer et al. 2020).

Environmental monitoring efforts at the Ringwood Superfund site have always prioritized groundwater testing out of concern over potential contamination migration towards a nearby potable water reservoir that serves over 3.5 million NJ residents (North Jersey District Water Supply Commission 2017). Soil investigations tended towards specific waste disposal areas, which have been cordoned off for remediation work (US Environmental Protection Agency 2014a, 2020). This compartmentalizing strategy may not fully recognize the history of the waste dumping practices, which saw refuse haphazardly strewn across the local landscape and beyond the confines of designated cleanup areas (Stodghill 2007). Although approximately 53,500 tons of paint sludge and associated soil have been removed, whether remediation efforts have meaningfully reduced soil contamination and associated health risks in areas regularly accessed by RLTC residents remains an ongoing debate.

To evaluate the efficacy of remediation efforts, this study investigates contamination in community soils on and around the Ringwood Superfund site by: (1) quantifying present-day heavy metal contamination and ecological risks using the geo-accumulation index (I_geo_) and ecological risk factor (E_r_); (2) identifying potential pollution sources through Positive Matrix Factorization (PMF) and UNMIX receptor models; and (3) estimating non-carcinogenic and carcinogenic risks via Monte Carlo simulations of the hazard index (HI) and total cancer risk (TCR). While this study focuses on the Ringwood Superfund site and the RLTC, similar stories are echoed nationwide. Thirteen percent of Superfund sites on the NPL are classified as being of *Native American Interest* and over 480,000 Native Americans live within three miles of a Superfund site (Azhar et al. 2025; Cade et al. 2025). However, the literature on pollution and Indigenous peoples is based largely on studies from a small number of communities (Fernández‐Llamazares et al. 2020, United Nations 2022). These patterns underscore the value of expanding research across a broader range of cultural, geographic, and exposure contexts. Against this backdrop, this study hopes to provide additional evidence to support efforts aimed at mitigating environmental health risks in other Native American communities facing similar challenges.

## Methods

### Study area

The Ringwood Superfund site is situated in the northern portion of the Borough of Ringwood, Passaic County, NJ, and encompasses 500 acres (2.02 km^2^) of forested areas, abandoned mine shafts, a portion of Ringwood State Park, a recycling center, and some 50 multigenerational homes built atop or adjacent to waste dumping sites. The site is bounded by Hope Mountain to the west and housed three major historical dumping sites: Cannon Mine Pit (CMP) to the south, Peters Mine Pit (PMP) to the north, and O’ Connor Disposal Area (OCDA), located south of PMP and along the eastern side of Peters Mine Rd (Borough of Ringwood Environmental Commission 2023, New Jersey Department of Health and Senior Services 2006). Ford Motor Company (hereinafter, Ford) and the Borough of Ringwood filled the tens-of-meters-deep CMP and PMP to ground surface with paint sludge, solvents, and municipal waste. OCDA initially served as a reservoir for mine tailings from wet ore processing operations before being repurposed by Ford as additional dumping ground after PMP had reached capacity. At present, large portions of the site have been fenced off, including the CMP, OCDA, and PMP areas (New Jersey Department of Health and Senior Services 2006, US Environmental Protection Agency 2014a, 2020).

### Sample collection and analysis

Soil samples were collected from five publicly accessible zones within the Ringwood Superfund site and from the area adjacent to its perimeter. Sampling zones were selected: (1) in partnership with community scientists from the RLTC, who provided insight into the frequency of visit of each zone and the general interest of the RLTC; and (2) based on relative distances from former dumping sites to investigate possible contaminant migration.

**Fig. 1:**
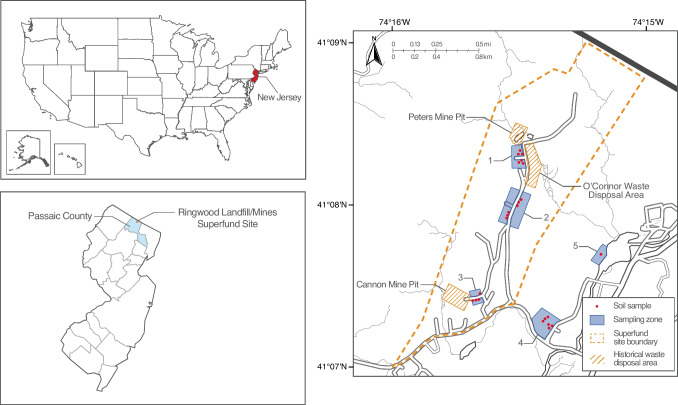
Location and layout of the ringwood superfund site and spatial distribution of soil samples

Within each zone, soil samples were obtained from two randomly-selected sub-locations, spaced 5 to 10 m apart. At each sub-location, three topsoil samples were collected from the upper 25 cm of the soil horizon, as this layer is most pertinent for considering exposure risks within the community. This hierarchical sampling design accounts for variability within and between sub‑locations in order to minimize bias from anomalous values and ensure that zone-level means are representative of real-life exposures. The spatial distribution of the soil samples is mapped in Fig. 1.

Samples were fractionated using U.S. Standard 2 mm (No. 10), followed by 63 µm (No. 230) sieves. The resulting 63-µm soil fraction was divided into 0.2-g triplicates to undergo acid digestion with a solution of ultra-pure nitric (HNO_3_), hydrochloric (HCl), and hydrofluoric (HF) acids in a 2:3:1 ratio (by volume). Acid-digested samples were analyzed by inductively coupled plasma-optical electron spectroscopy (ICP-OES) (Optima 4300 DV with AS 93plus autosampler, PerkinElmer, Shelton, CT) for the concentrations of the metals of concern at the site, i.e., antimony (Sb), arsenic (As), cadmium (Cd), cobalt (Co), chromium (Cr), copper (Cu), iron (Fe), lead (Pb), manganese (Mn), nickel (Ni), vanadium (V), and zinc (Zn). Metals with concentrations below the ICP-OES method detection limit were further analyzed with ICP-mass spectroscopy (ICP-MS) (NexION 2200 with S23 autosampler, PerkinElmer, Shelton, CT). ICP was calibrated with a five-point calibration curve, and QA/QC measures were taken to ensure quality data (Blake et al. 2015).

### Geo-accumulation index and ecological risk factor

To evaluate soil contamination, the Geo-accumulation Index (I_geo_, unitless) was calculated by comparing measured heavy metal concentrations to background levels in NJ using Eq. 1:

1 $${{I}_{geo}}_{i} = {\mathit{log}}_{2}\left(\frac{{C}_{i}}{{1.5\times B}_{i}}\right)$$

where ${C}_{i}$ and ${B}_{i}$ are the measured concentration and uncontaminated ambient background concentration in rural NJ soil (BEM Systems and Inc 2003a) of metal i, respectively.

Based on I_geo_ values, soil contamination can be classified as *uncontaminated* (I_geo_ ≤ 0), *uncontaminated to moderate* (0 < I_geo_ ≤ 1), *moderate* (1 < I_geo_ ≤ 2), *moderate to heavy* (2 < I_geo_ ≤ 3), *heavy* (3 < I_geo_ ≤ 4), *heavy to extreme* (4 < I_geo_ ≤ 5), or *extreme* (I_geo_ > 5) (Müller 1969).

I_geo_ is an extensively-cited reference framework, by virtue of its logarithmically-scaled pollution classes and its incorporation of an adjustment factor to account for lithogenic background fluctuation. However, it does not account for actual metal toxicity or bioavailability. Thus, the Ecological Risk Factor (E_r_, unitless) was also calculated to provide a more ecologically-focused evaluation of contamination levels using Eq. 2. Assessing both indices together provides complementary perspectives, with each addressing different aspects of contamination assessment that no single index can fully capture (Hoque et al. 2023; Kowalska et al. 2018).

2 $${{E}_{r}}_{i}=\frac{{C}_{i}}{{B}_{i}\times {{T}_{r}}_{i}}$$

where ${C}_{i}$ and ${B}_{i}$ are the measured concentration and uncontaminated ambient background concentration in rural NJ soil (BEM Systems and Inc 2003a) of metal i, respectively. ${{T}_{r}}_{i}$ is the metal-specific toxic response factor, which is equal to 1 for Mn, Sb, and Zn; 2 for Cr and V; 5 for Co, Cu, Ni, and Pb; 10 for As; and 30 for Cd.

Based on E_r_ values, potential ecological risk can be classified as *low* (E_r_ < 40), *moderate* (40 ≤ E_r_ < 80), *considerable* (80 ≤ E_r_ < 160), *high* (160 ≤ E_r_ < 320), or *very high* (E_r_ ≥ 320).

### Source apportionment

Two methods with different computational approaches, the Positive Matrix Factorization (PMF) Model and UNMIX were used to identify sources of soil heavy metals. PMF is a weighted least-squares factor analysis method that iteratively decomposes the concentration data into non-negative source profiles and source contribution matrices until the sum of squared residuals (*Q*) is minimized, weighted by the estimated uncertainty of each measurement. The incorporation of uncertainty estimates enables it to discern minor sources that could otherwise be masked by noise (US Environmental Protection Agency 2014b). However, its reliance on the user’s expert interpretation of diagnostic tests and user-controlled parameters, such as the number of factors and data uncertainty inputs may introduce some subjectivity into the analysis. UNMIX offers the advantage of being completely data-driven and assumption-free as it automatically estimates the number of sources and derives the source composition and contribution profiles directly from input data (US Environmental Protection Agency 2007). UNMIX robustly handles strong distinct source profiles but becomes less reliable in accurately quantifying sources with weak contributions as it struggles to resolve their signals from background noise (US Environmental Protection Agency 2007). Additional details for each method are provided in Table S1.

Each method was individually evaluated using *R*^2^, signal-to-noise ratio (S/N), and residuals. Results were also cross-compared between models to evaluate the consistency in source contribution profiles (Gong et al. 2024; Ma et al. 2023). Bootstrapping (BS) was conducted to examine the reproducibility of the PMF factors. Because PMF utilizes a bilinear factorization approach with minimal constraints built into the math, rotations of factor profiles (i.e. reallocations of elements across factors) can yield different but equally valid solutions to describe the underlying data structure. Thus, displacement (DISP) analysis was also conducted to examine the uniqueness of the resolved factors by determining how far each factor could be rotated before *Q* increases beyond a predefined tolerance (*dQ*_*max*_).

### Probabilistic human health risk assessment

Non-cancer and cancer health risks from soil and dust exposure through ingestion, inhalation, and dermal contact were evaluated for children (ages 1 to 11) and adults (ages 21 & above). Additionally, bioaccumulation of metals in wildlife tissues represents an indirect but potentially significant hazard for hunters and their families. This study’s concurrent Community Health Survey (CHS) (unpublished results) showed that approximatively 37% of the RLTC consumes venison from locally-hunted deer. Thus, exposure through consumption of game meat was evaluated as a fourth pathway. Following EPA Human Health Risk Assessment framework (Tetra Tech Incorporated 2025, US Environmental Protection Agency 1989), the chronic daily intake (CDI, mg·kg^−1^·day^−1^) from each pathway for each metal was first calculated using Eqs. 3–6:

3 $${{CDI}_{i}}_{ingestion, soil}= \frac{{C}_{i,soil}\times {10}^{-6}\times {IngR}_{soil}\times {EF}_{soil}\times ED }{BW\times AT}$$

4 $${{CDI}_{i}}_{inhalation}= \frac{{C}_{i,soil}\times InhR\times {EF}_{soil}\times ED }{BW\times AT\times PEF}$$

5 $${{CDI}_{i}}_{dermal}= \frac{{C}_{i,soil}\times {10}^{-6}\times SA\times AB{S}_{d}\times {EF}_{soil}\times ED }{BW\times AT\times AF}$$

6 $${{CDI}_{i}}_{ingestion, game}= \frac{{C}_{i,game}\times {10}^{-3}\times {IngR}_{game}\times {EF}_{game}\times ED }{BW\times AT}$$

where ${C}_{i,soil}$ and ${C}_{i,game}$ represent the soil concentration and the estimated concentration in game meat of metal i in mg·kg^−1^, respectively. For As only, the 60% default Relative Bioavailability value was used to adjust concentrations in order to not overestimate risks. BW (body weight, kg), ED (exposure duration, years), ${EF}_{game}$ (exposure frequency to game meat, days·year^−1^), and SA (skin surface area, cm^2^) were derived specifically for the RLTC from the CHS (Supplementary Methods S1). ${IngR}_{game}$ (intake of game meat, g·day^−1^) was derived from 24-h dietary recall data from the National Health and Nutrition Examination Survey (NHANES) from 2011 to 2018 (National Center for Health Statistics 2011–2018) (Supplementary Methods S2). The remaining parameters are defined in Tables S2-S3. Concentrations in game meat were estimated from soil concentrations using recommendations by Sample et al. (1998) (Sample et al. 1998) (Table S4). Since trivalent (Cr^III^) and hexavalent (Cr^VI^) chromium have different toxicity profiles, a 6:1 partitioning ratio was used to apportion Cr into Cr^III^ and Cr^VI^ as speciation assays were not performed (US Environmental Protection Agency 2024a).

Along with deterministic point values representing central tendency exposure (CTE) and reasonable maximum exposure (RME) scenarios (Table S3), Monte Carlo simulations (*N* = 10,000) generated distributions of possible CDI values. The resulting ranges of intake doses span across different “what-if” exposure scenarios, thereby capturing the inherent variability and uncertainty in exposure conditions (Panqing et al. 2023; US Environmental Protection Agency 2001). Non-cancer risk or hazard quotient (HQ, unitless) and cancer risk (CR, unitless) values from every exposure pathway were calculated for each metal using Eqs. 7 and 8, respectively:

7 $${HQ}_{i}=\sum_{j}^{{n}_{pathways}}\frac{{CDI}_{ij}}{{RfD}_{ij}}$$

8 $${CR}_{i}=\sum_{j}^{{n}_{pathways}}{CDI}_{ij}\times {CSF}_{ij}\times {ADAF}^{{\mathbb{I}}\left(i = {Cr}^{VI}\right)}$$

where ${RfD}_{ij}$ and ${CSF}_{ij}$ are reference dose and the cancer slope factor of metal i via pathway j, respectively; ADAF is the age-dependent adjustment factor for mutagenic carcinogens (applicable only to Cr^VI^), and is equal to 3 for children and 1 for adults (National Center for Environmental Asessment 2011). Metal-specific RfD and CSF are shown in Table S5.

Hazard index (HI, unitless) and total cancer risk (TCR, unitless) values associated with exposure to all 12 tested metals were ultimately obtained by summing the HQs and CRs from all metals as shown in Eqs. 9 and 10:

9 $$HI=\sum_{i}^{{n}_{metals}}{HQ}_{i}$$

10 $$TCR=\sum_{i}^{{n}_{carcinogens}}{CR}_{i}$$

### Statistical analysis

The statistical analysis and Monte Carlo simulations were performed using R software. Violin plots of the contamination indices, correlation matrices, radar plots of source contributions, human health risk probability curves were generated using R software. Source apportionment models were built with UNMIX 6.0 and PMF 5.0 (EPA, Washington, D.C.).

## Results

### Soil heavy metal concentrations

Heavy metal concentrations in the 23 soil samples collected from the study area are presented in Table 1, along with NJ ambient background levels and the EPA regional screening level (RSL) for residential soil, a health‑protective benchmark used to determine if further assessment of site‑related risks is warranted (US Environmental Protection Agency 2024c).

**Table 1:** Mean soil heavy metal concentrations (mg·kg^−1^) in and around the Ringwood Superfund site

Metal	Zone 1 (*n* = 6)	Zone 2 (*n* = 6)	Zone 3 (*n* = 4)	Zone 4 (*n* = 6)	Zone 5 (*n* = 1)	Aggregate (CV, %)	NJ Ambient^a,c^	RSL^b,d^
As	28.8	28.0	25.4	4.82	34.4	22.0 (64)	6.04	0.68
Cd	4.74	5.29	6.36	5.16	5.05	5.29 (24)	0.171	7.1
Co	19.2	15.4	13.9	10.1	12.9	14.7 (31)	6.45	23
Cr	42.8	60.0	45.8	40.2	37.1	46.9 (21)	19	0.95
Cu	64.9	43.3	41.8	22.4	32.8	42.8 (47)	18.5	3100
Fe	36,000	20,500	18,200	15,800	23,900	23,100 (50)	20,100	55,000
Mn	330	402	349	335	377	356 (33)	491	1800
Ni	53.2	45.7	36.4	45.9	58.7	46.7 (19)	12.7	840
Pb	346	34.9	36.0	518	198	249 (130)	35.1	200
Sb	4.73	3.83	3.32	4.10	2.90	4.01 (22)	0.395	31
V	234	124	90.3	94.9	128	140 (65)	33.5	460
Zn	112	117	81.6	117	101	109 (43)	72.1	23,000

*CV* coefficient of variation, *NJ* New Jersey, *RSL* Regional Screening Level

^a^Mean ambient levels in soils in rural NJ

^b^RSL for residential soil (target HQ = 1, target CR = 10^–6^)

^c^Data from BEM Systems Inc. (2003) (BEM Systems)

^d^Data from EPA (2024) (US Environmental Protection Agency 2024c)

Sitewide mean concentrations of the metals were in the order of Fe > Mn > Pb > V > Zn > Cr > Ni > Cu > As > Co > Cd > Sb. Levels of toxic metals such as As, Cd, Cr, Pb, Ni, and Sb all exceeded background levels in NJ. At all sampling zones, soil Cr levels ranging from (40.2 to 60.0) mg⋅kg^−1^ and As levels from (4.82 to 34.4) mg⋅kg^−1^ were consistently greater than their respective NJ backgrounds of 19.0 and 6.04 mg⋅kg^−1^, as well as RSLs of 0.950 and 0.680 mg⋅kg^−1^. Mean Cd levels in zone 3 (6.36 mg⋅kg^−1^) approached its RSL of 7.1 mg⋅kg^−1^, while levels of Ni ranging from (36.6 to 58.7) mg⋅kg^−1^ and Sb ranging from (3.32 to 4.73) mg⋅kg^−1^ remained below respective RSLs of 840 and 31 mg⋅kg^−1^ across all zones. Pb exhibited both high levels relative to NJ background of 35.1 mg⋅kg^−1^ and strong spatial differentiation (CV = 130%). Pb levels in zones 1, 4, and 5 were above or near the RSL of 200 mg⋅kg^−1^, averaging 346, 518, and 198 mg⋅kg^−1^, while levels in zones 2 and 3 averaged 34.9 and 36.0 mg⋅kg^−1^, respectively. Levels ranging from (22.4 to 64.9) mg⋅kg^−1^ for Cu, (15,800 to 36,000) mg⋅kg^−1^ for Fe, (330 to 402) mg⋅kg^−1^ for Mn, and (81.6 to 117) mg⋅kg^−1^ for Zn remained below corresponding RSLs across all five zones.

### Degree of heavy metal contamination and ecological risk

**Fig. 2:**
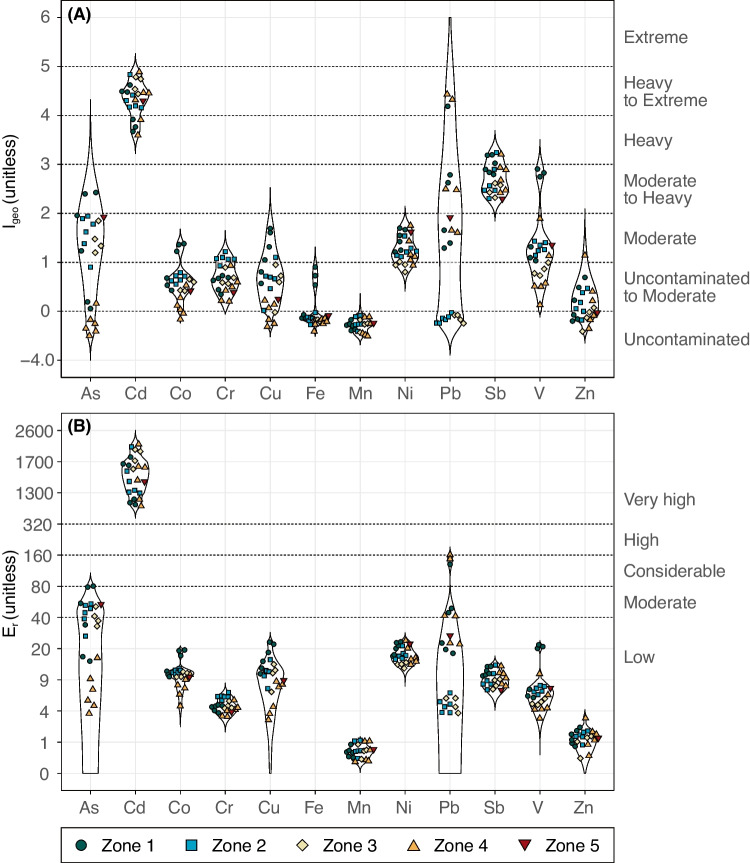
Distribution of soil heavy metal (**A**) geo-accumulation index and (**B**) ecological risk factor values. Abbreviations: I_geo_, geo-accumulation index; E_r_, ecological risk factor

I_geo_ and E_r_ were used to quantify and classify the contamination level and ecological risk of metals with respect to background levels in NJ. Figure 2 illustrates the distributions of I_geo_ and E_r_ values across the 23 soil samples.

For the contamination level of any given metal to be considered as *moderate* by I_geo_, its concentration must exceed the background level by a factor of three. By such criteria, every sample was classified as *uncontaminated* (I_geo_ ≤ 0) with Fe and Mn, with three samples from zone 1 (near PMP) showing a greater departure from NJ background levels (0 < I_geo_ ≤ 1). No samples were classified as being *uncontaminated* with Cd, Cr, Co (except two), Ni, Sb, and V. Soil samples were primarily classified as *uncontaminated to moderately contaminated* with Co and Cr. Ni and V contamination was generally *moderate* (1 < I_geo_ ≤ 2), with three samples from zone 1 reaching *moderate to heavy* V levels (2 < I_geo_ ≤ 3). Cd were consistently in the *heavy* (I_geo_ > 3) and *heavy to extreme* (I_geo_ > 4) contamination ranges. Contamination levels of As and Pb fluctuated greatly between sampling locations. Soil As contamination spanned across four different I_geo_ classifications, reaching *moderate to heavy* levels in zone 1. Pb contamination levels showed clear spatial differentiation across zones. While zones 2 and 3 were *uncontaminated*, zones 1, 4, and 5 showed *moderate* Pb contamination even at the lowest levels, reaching *heavy to extreme* levels at highest concentrations in zones 1 and 4.

Among the 12 metals assessed, only As, Cd, and Pb exceeded the *low* ecological risk threshold (E_r_ = 40). Levels of As and Pb exceeding this threshold generally fell within the *moderate* or *considerable* range (40 ≤ E_r_ < 160), though *heavy to extreme* Pb levels reached the *high* risk category (160 ≤ E_r_ < 320) in zone 4. Cd posed the highest ecological risks, with all 23 samples exhibiting *very high* E_r_ values (≥ 320).

### Source apportionment

#### Validation of receptor models

In PMF, convergence between *Q*_true_ and *Q*_robust_ was achieved at four factors, and additional factors produced only marginal reductions in *Q*_robust_ (US Environmental Protection Agency 2014b). Metal-specific *R*^*2*^ values for model’s goodness-of-fit ranged from 0.30 for Cr to 0.99 for Pb, with residuals generally falling within 3 standard deviations of 0. BS results showed that all four factors were successfully mapped in over 95% of bootstrap runs (*N* = 100) and no factor swaps were observed at *dQ*_*max*_ = 4, 8, 15, and 25 with DISP.

**Fig. 3:**
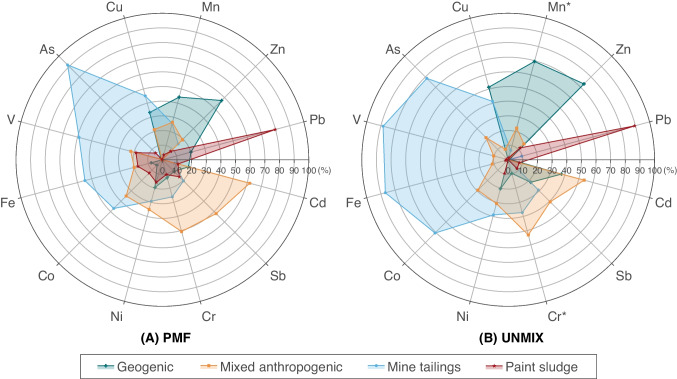
Source contribution profiles from (**A**) PMF and (**B**) UNMIX receptor models. Abbreviations: PMF, Positive Matrix Factorization. *Mn and Cr were omitted for UNMIX to generate a stable solution. Contributions to Mn and Cr levels in the UNMIX model were indirectly estimated by the program based on the resolved source profiles and remaining species

UNMIX also resolved four factors. Pearson correlation coefficient (*r*) values between corresponding source contribution profiles from UNMIX and PMF ranged from 0.86 and 0.96 (Fig. S1). Cr and Mn were excluded to achieve a solution that satisfies the minimum requirements for acceptance in UNMIX (min *R*^*2*^ > 0.8 and min S/N > 2.0) (US Environmental Protection Agency 2007), where min *R*^*2*^ was 0.96 and min S/N was 2.00, with residuals generally falling within 3 standard deviations of 0. Nonetheless, UNMIX includes post-hoc estimates of non-modeled species from each source, allowing Cr and Mn to be integrated in the final source contributions profile. Figure 3 shows the contributions to heavy metal levels from sources resolved by (A) PMF and (B) UNMIX models.

#### Source contribution profiles

**Fig. 4:**
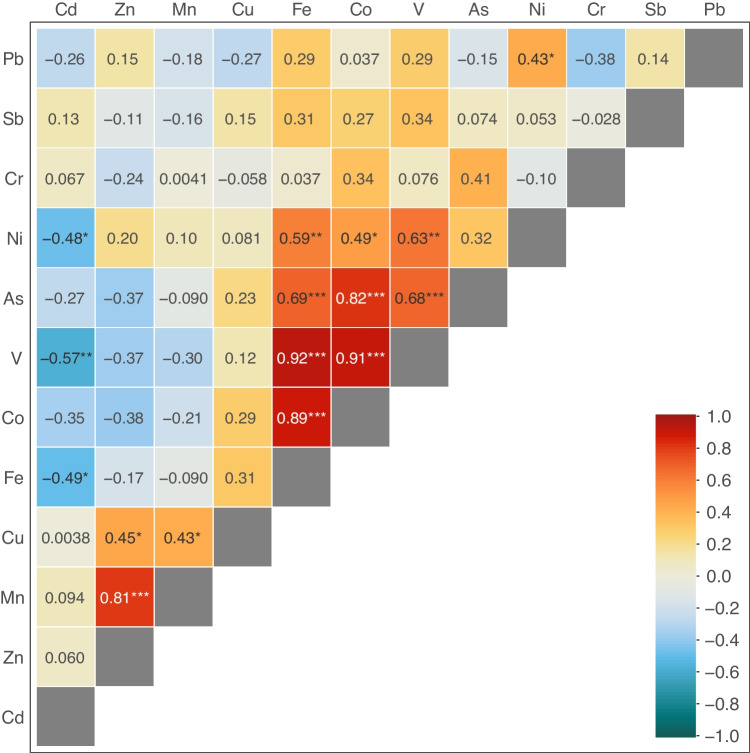
Pearson correlation coefficients between heavy metal concentrations (*N* = 23). Statistically significant correlations are indicated with *(*p*-value) ≤ 0.05; ** ≤ 0.01;*** ≤ 0.001

Source 1 was predominantly responsible for the presence of Cu, Mn, and Zn, with respective contributions of 33%, 44%, and 57% in PMF; and 51%, 69%, and 73% in UNMIX. The correlation matrix in Fig. 4 showed a strong Mn-Zn association (*r* = 0.81). Cu had moderate associations with Zn (*r* = 0.45) and Mn (*r* = 0.43), and a weak-to-moderate correlation with Fe (*r* = 0.31). Source 2 was dominated by highly collinear As, Co, Fe, and V (0.68 < *r* < 0.92). The respective loadings of As, Co, Fe, and V on source 2 were 92%, 47%, 55%, and 59% in PMF; and 79%, 71%, 87%, and 88% in UNMIX. PMF also attributed 47% of Cu, while UNMIX attributed 40% of Cu and Ni to this source. Source 3 was associated with weakly-correlated (0 < *r* < 0.2) Cd, Cr, Ni, and Sb. The respective fractions accounted for by source 3 were 62%, 51%, 35%, and 52% in PMF; and 54%, 53%, 31%, and 41% in UNMIX. PMF and UNMIX agreed on source 4 being the principal driver of soil Pb, deeming it responsible for 78% and 89% of the measured concentrations, respectively. Individually, PMF traced 15–20% of Fe, Ni, Sb, and V to this source. Source 4 contributed negligibly (< 15%) to the remaining metals compared to other sources.

### Monte Carlo simulations of health risk

**Fig. 5:**
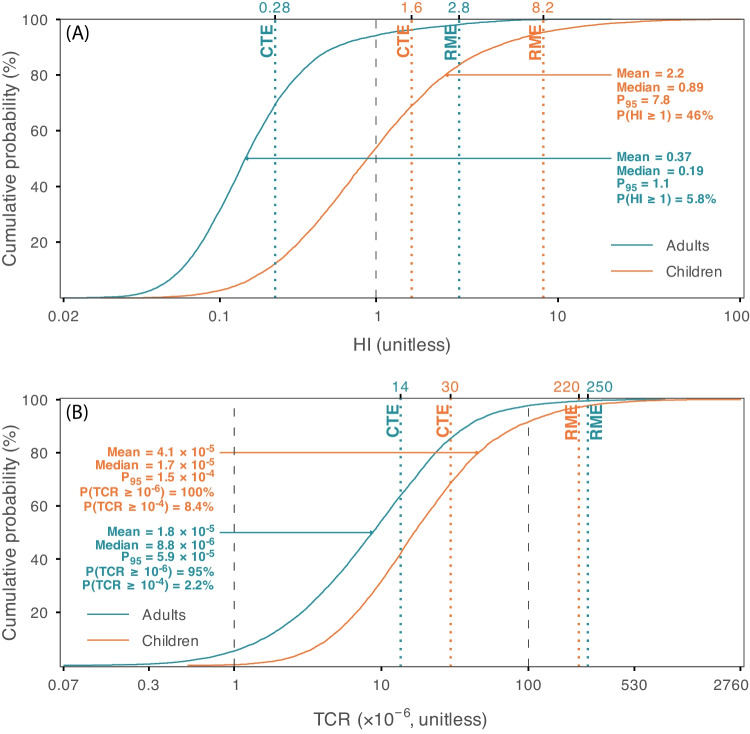
Cumulative distribution of Monte Carlo (**A**) hazard index and (**B**) total cancer risk values for children and adults. Abbreviations: CTE, central tendency exposure; HI, hazard index; RME: reasonable maximum exposure; P_95_: 95th percentile; TCR, total cancer risk. The dashed line in the HI plot at HI = 1 represents the EPA threshold for potential non-carcinogenic health effects. The dashed lines in the TCR plot at TCR = 10^–6^ and 10^–4^ represent the EPA thresholds for acceptable and unacceptable cancer risk, respectively

Non-cancer (HI) and cancer (TCR) risks associated with exposure to all 12 tested heavy metals in soil were evaluated for children and adults using Monte Carlo simulations. Figure 5 illustrates the cumulative distribution of calculated (A) HI and (B) TCR values.

HI and TCR values derived from CTE and RME scenarios served as benchmarks for evaluating Monte Carlo results. For both children and adults, CTE-based estimates fell between the mean and median, and RME‑based estimates fell between the 95th and 99th percentiles of the Monte Carlo HI and TCR distributions, indicating internal consistencies between deterministic and probabilistic approaches at both central and high‑end exposures.

HI values exceeding 1 suggest potential non-cancer risks that may require further evaluation or action (National Research Council 1994). 46% and 5.8% of the simulated HI values for children and adults exceeded 1, with corresponding means of 2.2 and 0.37. Nearly all TCR values exceeded the target cancer risk of 1 in 1,000,000 (TCR = 10^–6^), with 8.4% of children and 2.2% of adults facing cancer risks above the unacceptable threshold of 1 in 10,000 (TCR = 10^–4^), above which additional site intervention is warranted (US Environmental Protection Agency 1989). Mean TCR values were 4.1 × 10^−5^ for children and 1.8 × 10^−5^ for adults. Health risks were primarily driven by As and Cd (Table S6). HQ and CR from As exposure mainly reflected risks from soil ingestion and dermal contact, averaging 0.46 and 1.7 × 10^−5^ for children, and 0.069 and 1.1 × 10^−5^ for adults, respectively. Cd poses the highest non-cancer risk, with HQ averaging 0.65 and 0.11 for children and adults, respectively, largely due to game meat consumption (Table S6). Sensitivity analyses revealed exposure duration (ED, years) was the most significant driver of adult TCR with a Spearman correlation of 0.70. Game meat consumption frequency (EF_game_, days·year^−1^) was the most significant driver of adult HI and the second-most significant driver of child HI and TCR (Figs. S3 & S4) behind the soil ingestion rate (IngR_soil_, mg·day^−1^).

## Discussion

### Soil heavy metal contamination and ecological risk levels

When evaluating heavy metal concentrations in soil, it is important to consider absolute concentrations in the context of background levels, as many metals occur naturally due to parent material and geogenic processes. Levels of naturally-abundant Mn and Zn were two degrees of magnitude higher than their rarer but more toxic counterparts, such as Cd and Sb, but remained comparable to background levels and well below their respective RSLs, indicating low contamination and associated health risks. High Fe levels averaging 23,100 mg⋅kg^−1^ are consistent with the abundance of iron-rich quartz-oligoclase gneisses and amphibolites that constitute the local geology (Hotz 1953). However, the high CV at 50%, with mean levels reaching 36,000 mg⋅kg^−1^ in zone 1, suggests potential Fe enrichment by an additional source. I_geo_ effectively implicated anthropogenic sources in the Fe levels observed in zone 1 insofar as samples from this zone alone were classified as having Fe concentrations in the *uncontaminated to moderate* range.

The remaining metals exhibited I_geo_ values consistently above zero and each with its own distribution pattern. This variability constrained to the upper I_geo_ tiers suggests a multitude of anthropogenic sources with varying intensities in metal outputs. Ni, Cr, and Sb contamination were relatively tightly distributed across the site, reflecting diffuse, broad-impact sources indiscriminately impacting the study area. Conversely, sporadic instances of outstanding As and Pb levels signaled discrete pockets of heavy contamination resulting from point pollution sources or varying pollution dynamics across the site. I_geo_ denoted universal Cd contamination with levels beyond what is explicable by background fluctuations. In addition, E_r_ signaled that Cd contamination at the site is severe enough for substantial likelihood of adverse effects on local biota. Since Cd bioaccumulates extensively with minimal natural excretion (Peana et al. 2022; Satarug et al. 2017), failure to address contamination sustains its potential to move through food webs and ultimately reach humans.

### Soil heavy metal pollution sources

BS and DISP analysis results confirmed that PMF solution was robust and valid for source interpretation. Additionally, both PMF and UNMIX resolving four-factor solutions with high agreement among corresponding source contribution profiles (*r* < 0.7, *p* < 0.05) strengthened confidence in the source apportionment results and the interpretation of source identities. However, it is important to reiterate that Cr and Mn had to be omitted in order for UNMIX model to derive a stable solution and thus, contributions to these metals were extrapolated post hoc. While this facilitated comparisons with the PMF model, Cr and Mn source assignments in UNMIX carry greater uncertainty than those of the retained species.

Strong Mn-Zn association implied a shared source for these metals, hence their ascription to the same source. Cu’s electron configuration and ionic radius (~ 0.73 Å) confer a greater binding affinity to Fe, which may influence its mobility and explain its deviation from Mn and Zn (Peacock and Sherman 2004). Notwithstanding Cu hotspots coinciding spatially with elevated Fe, I_geo_ largely classified samples as being *uncontaminated to moderately contaminated* with Cu, Mn and Zn. These elements are naturally abundant in soil and their co-occurrence are generally attributed to the weathering of parent rocks and minerals (Linehan et al. 1989, Sanders 2003). There are no current or historical records of Cu or Zn mining, or agrochemical usage that could have explained some of the elevated levels observed (Alengebawy et al. 2021; Chen et al. 2022, 2008, Xu et al. 2025). Thus, source 1 was deemed the geogenic source.

Co-loadings of As, Co, Cu, and V onto source 2 underline the influence of Fe, commonly present in soil as Fe-(hydr)oxides, on a wide array of metals. The abundance and chemical stability of Fe-(hydr)oxide provide a consistent binding environment for many metals, effectively immobilizing them in the soil matrix via electrostatic or chemical adsorption, surface complexation, or coprecipitation (Peacock and Sherman 2004, Tian et al. 2024). Metals characterizing source 2, i.e., As, Co, Cu, and V, have a strong affinity for Fe and readily bind with it to co-occur at elevated levels in iron ores or iron-rich soils (Mikkonen et al. 2019; Peacock and Sherman 2004). Metal-laden ores become a hazard once hoisted to the surface and introduced to the upper soil levels through mining activities. Smelting byproducts, slag, tailings, and runoffs have been found to contaminate the soil around iron mining areas with the same metals as those in source 2 (Sloto 2011, Yang and Yang 2024). Evidently, I_geo_ showed peak As, Co, Cu, Fe, and V contamination in soil samples taken in zone 1 (near PMP), aligning with the documented history of iron ore mining and ironworks of the study area (New Jersey Department of Health and Senior Services 2006, Ransom 1966). Thus, source 2 was considered to be iron mine tailings or ironworks byproducts.

The presence of Cd portended source 3 to be of anthropogenic origin, as evidenced by I_geo_ values denoting *extreme* Cd contamination. However, the metals in this source exhibit correlation patterns inconsistent with any single emission source. Cd and Cr were discovered inside waste drums disposed in the area. Field reconnaissance surveys documented extremely corroded waste drums devoid of content, discarded vehicles, and steel‑belted tires throughout the site and near the sampling locations of this study (ARCADIS G&M, Inc. 2005). Cd and Cr were commonly used as pigments in car paints and, alongside Ni, are used in electroplating for vehicle exteriors (US Environmental Protection Agency 1996, Vitayavirasuk et al. 2005). Gradual weathering of discarded vehicles can leach these metals into the environment, as shown in studies on metals in soil surrounding scrapyards (Blake et al. 1987, Chicharro Martín et al. 1998). Ongoing vehicular traffic can also introduce Cd, Cr, Ni, and Sb into the soil via brake wear and tear or exhaust emissions, as reflected by high levels of these metals in this study’s roadside samples (zones 2 and 3) and in those from previous studies (Carrero et al. 2013). Because source 3 displayed partially overlapping fingerprints from multiple sources, it was categorized as a mixed anthropogenic source comprising disposed waste drums, vehicle‑related debris, and traffic emissions.

Elevated soil Pb is often tied to legacy contamination from lead-based paint or leaded gasoline (Battelle Memorial Institute 1998, Cook and Ni 2007). In this study, highest Pb levels were found in non-residential zones 1, 4, and 5. Zones 1 and 5 were inaccessible by motor vehicles and samples were taken away from built structures. Samples from zone 4 were taken 85 m away from the nearest road and 65 m away from the nearest building. Moreover, roadside samples from zones 2 and 3 showed no Pb elevation. Therefore, source 4 is unlikely to refer to lead-based paint or leaded gasoline, but rather appears unique to the Ringwood Superfund site. EPA documents reveal that Pb comprised over 6% of the paint sludge dumped in the study area (New Jersey Department of Health and Senior Services 2006). Zone 1’s adjacency to a historical dumping site (PMP) supports paint sludge as a plausible source. Although zones 4 and 5 lie outside Superfund boundaries, hydrological and topographical features of the site suggest potential contaminant migration. A surface body of water (Park Brook) drains zone 1 and discharges into zone 5. Local terrain shows elevation decreasing southeastward from the site. Zone 4, situated one km southeast of another paint sludge dumping site (OCDA) and within the same watershed that encompasses the study area, lies along surface water and shallow groundwater flow paths traversing the Ringwood Superfund site (Borough of Ringwood Environmental Commission 2023, New Jersey Department of Health and Senior Services 2006). This hydrological interconnectivity suggests that, despite the geographical partition, rainfall, snowmelt, surface runoffs, or groundwater may have mobilized paint beyond delineated boundaries, supporting paint sludge as the identity of source 4.

### Non-cancer and cancer risks from soil heavy metal exposure

Monte Carlo simulations revealed metal exposure could pose non-cancer and unacceptable cancer risks for both children and adults, with children facing disproportionately higher non-cancer risks than adults. Children are particularly vulnerable to non-occupational soil exposure due to hand-to-mouth behavior and frequent soil play, which increase their soil intake rate and exposure time to toxic contaminants in soil (Moya and Phillips 2014, Różański et al. 2021). Combined with a lower body weight and an immature metabolic and immune system, these factors greatly increase the contaminant dose per kilogram and associated risks for children. The convergence in TCR between children and adults is largely due to differences in exposure duration (ED). Because cancer risks build incrementally over time, longer lengths of exposure in adults increase their TCR even if acute effects (HI) may impact children more. Consequently, longer residence times on the Ringwood Superfund site may lead to cumulatively higher lifetime cancer risks.

Risk assessment results showed As predominantly driving risks through soil ingestion and dermal contact. Arsenic is well-absorbed through the gastrointestinal tract and chronic exposure causes distinct dermatological manifestations (Hong et al. 2014). Only total soil concentrations were measured without speciation or bioavailability analyses. While As concentrations were adjusted with a relative bioavailability factor, uncertainty remains regarding the fractions of the metal actually available for uptake. In addition to As, Cd contributed greatly to overall risks via the game meat consumption pathway. These findings corroborate E_r_ results identifying Cd as the dominant ecological hazard at the site due to its toxicity and persistence in the environment, and suggest that a revision of risk characterization at the site may be warranted.

## Conclusion

Results appeared to challenge official declarations that hazards have been removed and human exposure is under control at the Ringwood Mines/Landfill Superfund site. Arsenic, chromium, and lead were found at levels above their respective health-protective RSLs. Contamination indices (I_geo_ and E_r_) showed moderate to extreme exceedances of background levels in NJ and confirmed anthropogenic influence in the levels observed, with cadmium posing particularly high ecological risks. Despite remediation, PMF and UNMIX consistently identified source profiles linked to historical mining and waste disposal activities, suggesting that legacy contamination continues to influence present-day environmental conditions. Chronic uptake and bioaccumulation by local flora and fauna can facilitate trophic transfers of heavy metals to the local RLTC residents. Accordingly, Monte Carlo simulations revealed that heavy metal exposure posed non-cancer and cancer risks for both children and adults, with game meat consumption emerging as a potentially important exposure pathway. In light of these findings, this study highlights the importance of honoring cultural relationships with the land when characterizing exposure pathways and associated health risks in Native American communities.

## Supplementary Information

Below is the link to the electronic supplementary material.

ESM 1 (DOCX 731 KB)

## Data Availability

The data that support the findings of this study were obtained from the National Health and Nutrition Examination Survey (NHANES) and are publicly available from the Centers for Disease Control and Prevention (CDC) at https://wwwn.cdc.gov/nchs/nhanes/default.aspx. Raw data that support the findings of this study are available from the corresponding author, upon reasonable request.
