# Supplementary material for: Residual metal soil contamination and associated health risks in a native American community within a remediated Superfund site in New Jersey, USA
Source: Environ Sci Pollut Res Int. 2026 Jul 22;33(24):12305–18. doi: 10.1007/s11356-026-38049-4 (PMC13468744; doi:10.1007/s11356-026-38049-4)
Supplement: Supplementary file 1 [file 11356_2026_38049_MOESM1_ESM.docx]

**SUPPLEMENTARY MATERIALS FOR**

**Residual Metal Soil Contamination and Associated Health Risks in a Native American Community Within a Remediated Superfund Site in New Jersey, USA**

**Authors**:

Tri Huynh^1^, Eliane El Hayek^2^, Abdulmehdi Ali^3^, Tessa Hernandez^2^, Jania Zenon^1^, Connie Van Dunk^4^, Vincent Mann^4^, Esther Erdei^2^, Judith Zelikoff^1^

**Affiliations**:

^1^Division of Environmental Medicine, New York University Grossman School of Medicine, New York, NY 10010, United States

^2^Department of Pharmaceutical Sciences, College of Pharmacy, Health Sciences Center, University of New Mexico, Albuquerque, NM 87131, United States

^3^Department of Earth and Planetary Sciences, University of New Mexico, Albuquerque, NM 87131, United States

^4^The Ramapough Lunaape Turtle Clan, Ringwood, NJ, United States

**Corresponding** **Author**:

Judith Zelikoff

Division of Environmental Medicine

Department of Medicine

New York University Grossman School of Medicine

341 E 25^th^ Street

New York, NY 10010

Telephone: +1 (646) 754-5451

Email: [Judith.Zelikoff@nyulangone.org](mailto:Judith.Zelikoff@nyulangone.org)

TABLE OF CONTENTS

[Supplementary Methods	2](#_Toc229219934)

[Method S1 Derivation of game meat intake	2](#_Toc229219935)

[Method S2 Derivation of exposure frequency to game meat	2](#_Toc229219936)

[Supplementary Tables	3](#_Toc229219937)

[Table S1 Comparative Overview of Source Apportionment Models	3](#_Toc229219938)

[Table S2 Description and Probability Distribution of Input Parameters Used in Chronic Daily Intake Equations	4](#_Toc229219939)

[Table S3 Central Tendency Exposure (CTE) and Reasonable Maximum Exposure (RME) Input Parameters Used in Chronic Daily Intake Equations	5](#_Toc229219940)

[Table S4 Estimate Models of Bioaccumulation of Metal Concentration in Soil by Herbivorous Small Mammals	6](#_Toc229219941)

[Table S5 Metal-Specific Reference Doses and Cancer Slope Factors	7](#_Toc229219942)

[Table S6 Non-Cancer Risk (Hazard Quotient [HQ], unitless) of Soil Heavy Metals via Soil Ingestion, Game Meat Ingestion, Dust Inhalation, Dermal Contact	8](#_Toc229219943)

[Table S7 Cancer Risk (Cancer Risk [CR], unitless) of Soil Heavy Metals via Soil Ingestion, Game Meat Ingestion, Dust Inhalation, Dermal Contact	9](#_Toc229219944)

[Supplementary Figures	10](#_Toc229219945)

[Figure S1 Spearman Rank Correlation Between Source Contributions from PMF and UNMIX Models	10](#_Toc229219946)

[Figure S2 Sensitivity Analysis (Spearman Rank Correlation) of Input Parameters of Hazard Index (HI) for (A) Children and (B) Adults	11](#_Toc229219947)

[Figure S3 Sensitivity Analysis (Spearman Rank Correlation) of Input Parameters of Total Cancer Risk (TCR) for (A) Children and (B) Adults	12](#_Toc229219948)

# Supplementary Methods

## Method S1 Derivation of game meat intake

24-hour dietary recall data from the National Health and Nutrition Examination Survey (NHANES) 2011–2018 were used to estimate the mean ingestion rate of game meat among consumers.

Dietary intake data contained in the “Day 1 Individual Foods” files from four consecutive two-year NHANES survey cycles were downloaded and merged, in accordance with National Center for Health Statistics analytic guidelines for pooled analyses^1^. The second day of dietary recall is generally not used to estimate means. To combine multiple cycles, the dietary day-one sample weights (WTDRD1) were divided by the number of cycles (i.e., four) to create an adjusted eight-year weight.

Game meat consumption was identified through U.S. Department of Agriculture food codes and associated food descriptions. Calculations incorporated the NHANES survey design, which specified strata and primary sampling units along with the combined dietary weight. All point estimates and variance estimates were computed using the *survey* (v4.4.8; Lumley, 2025) package in R, which applies Taylor series linearization to obtain appropriate standard errors and confidence intervals^2^. After defining the survey design, analyses were restricted to the subset of respondents who reported consuming game meat within the recall period (consumers only), and the ingestion rate per consumption day was calculated as the mean intake (g·consumption day^-1^) among consumers.

## Method S2 Derivation of exposure frequency to game meat

Preliminary from this study’s concurrent Community Health Survey (unpublished results) were analyzed to estimate annual exposure frequency to locally hunted game meat (consumption days per year). Participants were asked (1) whether they consumed game meat in the past 12 months, with response options converted to estimated annual consumption days as follows: "Everyday" = 365 days; "More than once a week but not every day" = 156 days (3 days/week · 52 weeks); "Once a week" = 52 days (1 day/week · 52 weeks); "More than once a month, but not every week" = 24 days (1 day/2 weeks · 52 weeks); "Once a month" = 12 days (1 day/month · 12 months); "Once every few months" = 4 days (1 day/3 months · 12 months). Participants were also asked to report the proportion of their game meat that was hunted inside the Ringwood Superfund site, with categories converted to percentages: "All" = 100%; "Most" = 75%; "Half" = 50%; "Some" = 25%; "None" = 0%. Exposure frequency to game meant (${EF}_{game}$) was then calculated for each respondent as ${EF}_{game}=D \times P$ where $D$ is the estimated annual consumption days and $P$ is the proportion of locally hunted game meat.

# Supplementary Tables

## Table S1 Comparative Overview of Source Apportionment Models

| Method | Equation | Term Definitions | Strengths | Limitations |
| --- | --- | --- | --- | --- |
| EPA UNMIX (UNMIX)^a^ | $C_{ij}=\sum_{k}^{n_{factors}} F_{ik}\times S_{kj}$ | $C_{ij}$ – concentration of metal $i$ in sample $j$  $F_{ik}$ – fraction of metal $i$ in source $k$  $S_{kj}$ – contribution of source $k$ to sample $j$ | - Friendly user interface - Assumption-free, data-driven, automatic estimation of number of sources - Identification of outliers with suggestions for exclusion - Handling of small data gaps - Non-negativity constraints | - Linearity assumption vs. real-world dynamics - Reduced effectiveness in presence of overlapping or weak sources - Sensitivity to data quality (outliers, scale differences) - Lack of consideration for measurement uncertainty |
| Positive Matrix Factorization (PMF)^b^ | $C_{ij}=\sum_{k}^{n_{factors}} {F_{ik}\times G}_{kj}+E_{ij}$ | $C_{ij}$ – concentration of metal $i$ in sample $j$  $G_{kj}$ – contribution of source $k$ to sample $j$  $F_{ik}$ – fraction of metal $i$ in source $k$  $E_{ij}$– the portion of metal $i$ in sample $j$ unexplained by the model after minimizing $Q=\sum_{i}^{n_{metals}} \sum_{j}^{n_{factors}} \left( \frac{E_{ij}}{U_{ij}} \right)^{2}$, where $U_{ij}$ is the uncertainty of metal $i$ in sample $j$ | - Incorporation of uncertainties - Resolution of complex mixtures of overlapping sources - Flexibility in handling sources of varying strengths - Handling of data gaps - Non-negativity constraints | - Model complexity - Sensitivity to data quality (outliers, scale differences) - Sensitivity to selection of number of sources - Dependence on accuracy of input uncertainties - Rotational ambiguity leading to mathematically equivalent but vastly different source profiles |
| From ^a^U.S. EPA (2007)^3^. ^b^U.S. EPA (2014)^4^. | | | | |

## Table S2 Description and Probability Distribution of Input Parameters Used in Chronic Daily Intake Equations

| Abbreviation | Pathway | Description | Unit | Distribution | Value | |
| --- | --- | --- | --- | --- | --- | --- |
|  |  |  |  |  | Children | Adults |
| $InhR$ | Inhalation | Inhalation rate | m^3^·day^-1^ | Point | 13^a^ | 16^a^ |
| $PEF$ | Inhalation | Particulate emission factor | m^3^·kg^-1^ | Point | 1.36 × 10^9b^ | |
| ${IngR}_{soil}$ | Ingestion | Soil ingestion rate | mg·day^-1^ | Lognormal | 100, 200^b^ | 50, 100^b^ |
| ${IngR}_{game}$ | Ingestion | Game meat intake | g·consumption day^-1^ | Gamma | 89, 255^c^ | |
| ${EF}_{game}$ | Ingestion | Exposure frequency to game meat | consumption days·year^-1^ | Empirical | 0, 12, 98, 156^d^ | |
| $SA$ | Dermal | Skin surface area | cm^2^ | Lognormal (children); Empirical (adults) | 3560, 6020^a^ | 3460, 5110, 6880, 8030^d^ |
| $AF$ | Dermal | Adherence factor to skin | mg·cm^-2^ | Point | 0.2^b^ | 0.07^b^ |
| ${ABS}_{d}$ | Dermal | Dermal absorption fraction | unitless | Point | Metal-specific (0.03 for As; 0.001, otherwise) | |
| ${EF}_{soil}$ | All (soil only) | Exposure frequency to soil | days·year^-1^ | Lognormal | 250, 350^b^ | |
| $ED$ | All | Exposure duration | years | Lognormal (children); Empirical (adults) | 6, 9^b^ | 1, 25, 56, 70^d^ |
| $BW$ | All | Body weight | kg | Lognormal (children); Empirical (adults) | 22, 50^a^ | 50, 89, 130, 160^d^ |
| $AT$(HI) | All | Averaging time for HI | days | Lognormal (children); Empirical (adults) | $ED$ x 365^b^ | |
| $AT$ (TCR) | All | Averaging time for TCR | days | Point | 70^b^ | |
| Abbreviations: CTE: central tendency exposure; RME: reasonable maximum exposure; HI: hazard index; TCR: total cancer risk.  (a1, a2) defines the mean and 95^th^ percentile of the lognormal distribution, or the shape and scale parameters of the gamma distribution.  (b1, b2, b3, b4) defines the minimum, mean, 95^th^ percentile, and maximum of the empirical data from this study’s concurrent Community Health Survey (unpublished results).  ^a^Data derived from age-group (ages 1 to 11 for children and ages 21 or higher for adults) means and 95^th^ percentiles weighted by sample size in the U.S. EPA Exposure Factor Handbook^5^.  ^b^Data from U.S. EPA Exposure Factor Handbook^5^.  ^c^Data derived from National Health and Nutrition Examination Survey (NHANES) 24-hour dietary recall (2011-2018)^6^.  ^d^Preliminary data derived from this study’s concurrent Community Health Survey (unpublished results). | | | | | | |

| Abbreviation | Pathway | Description | Unit | CTE | |  | RME | |
| --- | --- | --- | --- | --- | --- | --- | --- | --- |
|  |  |  |  | Children | Adults |  | Children | Adults |
| $InhR$ | Inhalation | Inhalation rate | m^3^·day^-1^ | 13^a^ | 16^a^ |  | 13^a^ | 16^a^ |
| $PEF$ | Inhalation | Particulate emission factor | m^3^·kg^-1^ | 1.36 × 10^9b^ | |  | 1.36 × 10^9b^ | |
| ${IngR}_{soil}$ | Ingestion | Soil ingestion rate | mg·day^-1^ | 60^b^ | 50^b^ |  | 200^b^ | 100^b^ |
| ${IngR}_{game}$ | Ingestion | Game meat ingestion rate | g·consumption day^-1^ | 89^c^ | |  | 255^c^ | |
| ${EF}_{game}$ | Ingestion | Exposure frequency to game meat | consumption days·year^-1^ | 12^d^ | |  | 98^d^ | |
| $SA$ | Dermal | Skin surface area | cm^2^ | 3560^a^ | 5110^a^ |  | 6020^d^ | 6880^d^ |
| $AF$ | Dermal | Adherence factor to skin | mg·cm^-2^ | 0.2^b^ | 0.07^b^ |  | 0.2^b^ | 0.07^b^ |
| ${ABS}_{d}$ | Dermal | Dermal absorption fraction | unitless | Metal-specific (0.03 for As; 0.001, otherwise) | | | | |
| ${EF}_{soil}$ | All (soil only) | Exposure frequency to soil | days·year^-1^ | 250^b^ | |  | 350^b^ | |
| $ED$ | All | Exposure duration | years | 6^b^ | 25^d^ |  | 9^b^ | 56^d^ |
| $BW$ | All | Body weight | kg | 22^b^ | 89^d^ |  | 50^b^ | 130^d^ |
| $AT$(HI) | All | Averaging time for HI | days | $ED$ x 365^b^ | |  | $ED$ x 365^b^ | |
| $AT$ (TCR) | All | Averaging time for TCR | days | 70^b^ | |  | 70^b^ | |
| Abbreviations: CTE: central tendency exposure; RME: reasonable maximum exposure; HI: hazard index; TCR: total cancer risk.  ^a^Data derived from age-group (ages 1 to 11 for children and ages 21 or higher for adults) means and 95^th^ percentiles weighted by sample size in the U.S. EPA Exposure Factor Handbook^5^.  ^b^Data from U.S. EPA Exposure Factor Handbook^5^.  ^c^Data derived from National Health and Nutrition Examination Survey (NHANES) 24-hour dietary recall (2011-2018)^6^.  ^d^Preliminary data derived from this study’s concurrent Community Health Survey (unpublished results). | | | | | | | | |

## Table S3 Central Tendency Exposure (CTE) and Reasonable Maximum Exposure (RME) Input Parameters Used in Chronic Daily Intake Equations

## Table S4 Estimate Models of Bioaccumulation of Metal Concentration in Soil by Herbivorous Small Mammals

| Metal | Recommended method | Equation |
| --- | --- | --- |
| As | Trophic-group regression | $\ln\left( C_{game} \right)= -5.6531+1.1382\times\ln\left( C_{soil} \right)$ |
| Cd | Trophic-group regression | $\ln\left( C_{game} \right)=-1.2571+0.4723\times\ln\left( C_{soil} \right)$ |
| Co | Median general UF | $C_{game}=0.0205\times C_{soil}$ |
| Cr | Median trophic-group UF | $C_{game}=0.0884\times C_{soil}$ |
| Cu | Median trophic-group UF | $C_{game}=0.1086\times C_{soil}$ |
| Fe | Trophic-group regression | $\ln\left( C_{game} \right)= -0.4758+0.6207\times\ln\left( C_{soil} \right)$ |
| Mn | Median trophic-group UF | $C_{game}=0.0156\times C_{soil}$ |
| Ni | General regression | $\ln\left( C_{game} \right)= -0.2462+0.4658\times\ln\left( C_{soil} \right)$ |
| Pb | Trophic-group regression | $\ln\left( C_{game} \right)= -0.6114+0.5181\times\ln\left( C_{soil} \right)$ |
| Sb | None | *N/A* |
| V | Median trophic-group UF | $C_{game}=0.0129\times C_{soil}$ |
| Zn | General regression | $\ln\left( C_{game} \right)= 4.4713+0.0738\times\ln\left( C_{soil} \right)$ |
| Abbreviations: UF, uptake factor; $C_{game}$, concentration in herbivorous mammal (mg·kg^-1^); $C_{soil}$, measured concentration in soil (mg·kg^-1^); N/A, not applicable.  From Sample et al., (1988)^7^. | | |

## Table S5 Metal-Specific Reference Doses and Cancer Slope Factors

|  | Reference Dose (RfD, mg·kg^−1^·day^−1^) | | |  | Cancer Slope Factor (CSF, (mg·kg^−1^·day^−1^)^−1^) | | |
| --- | --- | --- | --- | --- | --- | --- | --- |
| Metal | Oral | Inhalation | Dermal |  | Oral | Inhalation | Dermal |
| As | 3.0 × 10^-4^ | 4.3 × 10^-6^ | 3.0 × 10^-4^ |  | 1.5 | 15 | 1.5 |
| Cd | 1.0 × 10^-4^ | 2.9 × 10^-6^ | 2.5 × 10^-6^ |  | 0.5^a^ | 6.3 | 15 |
| Co | 3.0 × 10^-4^ | 1.7 × 10^-6^ | 3.0 × 10^-4^ |  |  | 32 |  |
| Cr(III) | 1.5 | 1.7 × 10^-6^ | 2.0 × 10^-2^ |  |  |  |  |
| Cr(VI) | 9.0 × 10^-4^ | 8.5 × 10^-6^ | 2.3 × 10^-5^ |  | 0.16 | 39 | 6.4 |
| Cu | 4.0 × 10^-2^ |  | 4.0 × 10^-2^ |  |  |  |  |
| Fe | 0.70 |  | 0.70 |  |  |  |  |
| Mn | 2.4 × 10^-2^ | 1.4 × 10^-6^ | 9.6 × 10^-4^ |  |  |  |  |
| Ni | 1.1 × 10^-2^ | 5.7 × 10^-6^ | 4.4 × 10^-4^ |  |  | 0.91 |  |
| Pb | 3.5 × 10^-3b^ | 3.5 × 10^-3b^ | 5.3 × 10^-4b^ |  | 8.5 × 10^-3^ | 4.2 × 10^-2^ | 8.5 × 10^-3^ |
| Sb | 4.0 × 10^-4^ | 8.5 × 10^-5^ | 6.0 × 10^-5^ |  |  |  |  |
| V | 9.0 × 10^-3^ | 2.0 × 10^-6^ | 2.3 × 10^-4^ |  |  | 29 |  |
| Zn | 0.30 |  | 0.30 |  |  |  |  |
| Data from U.S. EPA^8^, unless noted otherwise.  The inhalation reference dose (RfD_inh,_ mg·kg^−1^·day^−1^) was calculated from inhalation reference concentration (RfC, mg·m^-3^) using ${RfD}_{inh}=\frac{RfC \times InhR}{BW}$ and the inhalation cancer slope factor (CSF_inh_, (mg·kg^−1^·day^−1^)^−1^) was calculated from the inhalation unit risk (IUR, (µg·m^-3^)^−1^) using ${CSF}_{inh}=\frac{IUR \times BW\times1000 \mu g/mg}{InhR}$, where $BW$ is the default adult body weight^9^ (70 kg) and $InhR$ is the default adult inhalation rate^9^ (20 m^3^·day^-1^).  The dermal reference dose (RfD_derm,_ mg·kg^−1^·day^−1^) and cancer slope factor (CSF_derm_, (mg·kg^−1^·day^−1^)^−1^) were derived from their oral counterparts using ${RfD}_{derm}={RfD}_{oral}\times{ABS}_{GI}$ and ${CSF}_{derm}={{CSF}_{oral}}/{{ABS}_{GI}}$, where ${ABS}_{GI}$ is the gastrointestinal absorption factor (unitless), which is equal to 0.013 for Cr(III), 0.025 for Cd and Cr(VI), 0.026 for V, 0.040 for Ni, 0.150 for Sb, and 1.00 for the remaining metals^10^.  ^a^From Shi et al., (2025)^11^.  ^b^From Wang et al., (2022)^12^. | | | | | | | |

|  | Children | | | | |  | Adults | | | | |
| --- | --- | --- | --- | --- | --- | --- | --- | --- | --- | --- | --- |
| Metal | HQ_ingestion,soil_ | HQ_ingestion, game_ | HQ_inhalation_ | HQ_dermal_ | HI |  | HQ_ingestion,soil_ | HQ_ingestion, game_ | HQ_inhalation_ | HQ_dermal_ | HI |
| As | 0.30 | 0.050 | 3.8 × 10^-3^ | 0.11 | 0.46 |  | 0.048 | 9.6 × 10^-3^ | 9.1 × 10^-4^ | 0.010 | 0.069 |
| Cd | 0.13 | 0.46 | 8.3 × 10^-4^ | 0.061 | 0.65 |  | 0.021 | 0.087 | 2.0 × 10^-4^ | 6.0 × 10^-3^ | 0.11 |
| Co | 0.12 | 0.074 | 3.8 × 10^-3^ | 1.4 × 10^-3^ | 0.20 |  | 0.019 | 0.014 | 9.2 × 10^-4^ | 1.4 × 10^-4^ | 0.034 |
| Cr(III) | 6.7 × 10^-5^ | 1.7 × 10^-4^ | 1.0 × 10^-3^ | 6.0 × 10^-5^ | 1.3 × 10^-3^ |  | 1.1 × 10^-5^ | 3.3 × 10^-5^ | 2.5 × 10^-4^ | 5.9 × 10^-6^ | 3.0 × 10^-4^ |
| Cr(VI) | 0.018 | 0.050 | 3.5 × 10^-4^ | 8.7 × 10^-3^ | 0.077 |  | 2.9 × 10^-3^ | 9.6 × 10^-3^ | 8.4 × 10^-5^ | 8.4 × 10^-4^ | 0.013 |
| Cu | 2.7 × 10^-3^ | 8.6 × 10^-3^ |  | 3.1 × 10^-5^ | 0.011 |  | 4.2 × 10^-4^ | 1.7 × 10^-3^ |  | 3.0 × 10^-6^ | 2.1 × 10^-3^ |
| Fe | 0.082 | 0.033 |  | 9.6 × 10^-4^ | 0.12 |  | 0.013 | 6.2 × 10^-3^ |  | 9.4 × 10^-5^ | 0.019 |
| Mn | 0.036 | 0.017 | 0.011 | 0.011 | 0.075 |  | 5.8 × 10^-3^ | 3.2 × 10^-3^ | 2.7 × 10^-3^ | 1.1 × 10^-3^ | 0.013 |
| Ni | 1.00E-02 | 0.031 | 3.7 × 10^-3^ | 3.1 × 10^-3^ | 0.049 |  | 1.7 × 10^-3^ | 5.9 × 10^-3^ | 8.7 × 10^-4^ | 3.0 × 10^-6^ | 8.8 × 10^-3^ |
| Pb | 0.18 | 0.17 | 1.1 × 10^-4^ | 0.014 | 0.37 |  | 0.028 | 0.031 | 2.7 × 10^-5^ | 1.3 × 10^-3^ | 0.061 |
| Sb | 0.025 | 0.037 | 2.1 × 10^-5^ | 1.9 × 10^-3^ | 0.064 |  | 4.0 × 10^-3^ | 7.1 × 10^-3^ | 5.0 × 10^-6^ | 1.9 × 10^-6^ | 0.011 |
| V | 0.038 | 0.014 | 0.031 | 0.017 | 0.10 |  | 6.1 × 10^-3^ | 2.8 × 10^-3^ | 7.5 × 10^-3^ | 1.7 × 10^-3^ | 0.018 |
| Zn | 8.8 × 10^-4^ | 0.03 |  | 1.1 × 10^-5^ | 0.031 |  | 1.4 × 10^-4^ | 5.7 × 10^-3^ |  | 1.0 × 10^-6^ | 5.90 × 10^-3^ |
| **Total** | **0.94** | **0.97** | **0.056** | **0.23** | **2.2** |  | **0.15** | **0.18** | **0.013** | **0.022** | **0.37** |
| Abbreviations: HQ, hazard quotient; HI, hazard index.  Values are mean HQ and HI values from 10,000 Monte Carlo simulations. | | | | | | | | | | | |

## Table S6 Non-Cancer Risk Assessment (Hazard Quotient [HQ], unitless) via Soil Ingestion, Game Meat Ingestion, Dust Inhalation, Dermal Contact

## Table S7 Cancer Risk Assessment (Cancer Risk [CR], unitless) via Soil Ingestion, Game Meat Ingestion, Dust Inhalation, Dermal Contact

|  | Children | | | | |  | Adults | | | | |
| --- | --- | --- | --- | --- | --- | --- | --- | --- | --- | --- | --- |
| Metal | CR_ingestion,soil_ | CR_ingestion, game_ | CR_inhalation_ | CR_dermal_ | TCR |  | CR_ingestion,soil_ | CR_ingestion, game_ | CR_inhalation_ | CR_dermal_ | TCR |
| As | 1.1 × 10^-5^ | 1.9 × 10^-6^ | 1.8 × 10^-8^ | 4.1 × 10^-6^ | 1.7 × 10^-5^ |  | 7.6 × 10^-6^ | 1.5 × 10^-6^ | 1.8 × 10^-8^ | 1.6 × 10^-6^ | 1.1 × 10^-5^ |
| Cd | 5.6 × 10^-7^ | 2.0 × 10^-6^ | 1.1 × 10^-9^ | 2.0 × 10^-7^ | 2.7 × 10^-6^ |  | 3.7 × 10^-7^ | 1.5 × 10^-6^ | 1.1 × 10^-9^ | 7.9 × 10^-8^ | 2.0 × 10^-6^ |
| Co |  |  | 1.6 × 10^-8^ |  | 1.6 × 10^-8^ |  |  |  | 1.5 × 10^-8^ |  | 1.5 × 10^-8^ |
| Cr^a^ | 4.8 × 10^-6^ | 1.3 × 10^-5^ | 1.8 × 10^-7^ | 2.2 × 10^-6^ | 2.0 × 10^-5^ |  | 1.0 × 10^-6^ | 3.3 × 10^-6^ | 5.9 × 10^-8^ | 3.0 × 10^-7^ | 4.7 × 10^-6^ |
| Cu |  |  |  |  |  |  |  |  |  |  |  |
| Fe |  |  |  |  |  |  |  |  |  |  |  |
| Mn |  |  |  |  |  |  |  |  |  |  |  |
| Ni |  |  | 1.4 × 10^-9^ |  | 1.4 × 10^-9^ |  |  |  | 1.4 × 10^-9^ |  | 1.4 × 10^-9^ |
| Pb | 4.6 × 10^-7^ | 4.4 × 10^-7^ | 3.5 × 10^-10^ | 5.3 × 10^-9^ | 9.1 × 10^-7^ |  | 2.9 × 10^-7^ | 3.2 × 10^-7^ | 3.4 × 10^-10^ | 2.1 × 10^-9^ | 6.1 × 10^-7^ |
| Sb |  |  |  |  |  |  |  |  |  |  |  |
| V |  |  | 1.3 × 10^-7^ |  | 1.3 × 10^-7^ |  |  |  | 1.3 × 10^-7^ |  | 1.3 × 10^-7^ |
| Zn |  |  |  |  |  |  |  |  |  |  |  |
| **Total** | **1.7 × 10^-5^** | **1.7 × 10^-5^** | **3.5 × 10^-7^** | **6.5 × 10^-6^** | **4.1 × 10^-5^** |  | **9.3 × 10^-6^** | **6.6 × 10^-6^** | **2.2 × 10^-7^** | **2.0 × 10^-6^** | **1.8 × 10^-5^** |
| Abbreviations: CR, cancer risk; TCR, total cancer risk.  Values are mean CR and TCR values from 10,000 Monte Carlo simulations.  ^a^Cr was assumed to be all Cr(VI). | | | | | | | | | | | |

# Supplementary Figures

## Figure S1 Spearman Rank Correlation Between Source Contributions from PMF and UNMIX Models

Abbreviations: PMF, Positive Matrix Factorization.

Statistically significant correlations are indicated with *(*p*-value) ≤ 0.05; ** ≤ 0.01;*** ≤ 0.001; ns > 0.05.

## Figure S2 Sensitivity Analysis (Spearman Rank Correlation) of Input Parameters of Hazard Index (HI) for (A) Children and (B) Adults

Abbreviations: HI, hazard index.

Bar graphs are labeled with the proportion of HI variance explained by the input parameters.

## Figure S3 Sensitivity Analysis (Spearman Rank Correlation) of Input Parameters of Total Cancer Risk (TCR) for (A) Children and (B) Adults

Abbreviations: TCR, total cancer risk.

Bar graphs are labeled with the proportion of TCR variance explained by the input parameters.

References

1.	Centers for Disease Control and Protection, Statistics NCfH. NHANES Tutorials. National Center for Health Statistics. Accessed November 10, 2025. <https://wwwn.cdc.gov/nchs/nhanes/tutorials/default.aspx>

2.	*survey: Analysis of Complex Survey Samples*. Version R package version 4.4.2. 2025.

3.	EPA Unmix 6.0 Fundamentals & User Guide (U.S. Environmental Protection Agency) (2007).

4.	EPA Positive Matrix Factorization (PMF) 5.0 Fundamentals and User Guide (U.S. Environmental Protection Agency) (2014).

5.	National Center for Environmental A, United States. Environmental Protection Agency. Office of Research and D. *Exposure factors handbook : 2011 edition*. National Center for Environmental Assessment, Office of Research and Development, U.S. Environmental Protection Agency; 2011.

6.	Centers for Disease Control and Prevention, National Center for Health Statistics. Data from: National Health and Nutrition Examination Survey Data. 2011-2018. *Hyattsville, MD*.

7.	Development and validation of bioaccumulation models for small mammals (Lockheed Martin Energy Systems, Inc.) (1998).

8.	Regional Screening Level (RSL) Resident Soil Table (TR=1E-06, HQ=1) November 2024 (U. S. Environmental Protection Agency) (2024).

9.	RSEI Methodology Document for Version 2.3.12 Technical Appendix A - Toxicity Weights for TRI Chemicals and Chemical Categories (U.S. Environmental Protection Agency) (2024).

10.	1 Risk Assessment Guidance for Superfund: Volume I - Human Health

Evaluation Manual (Part E, Supplemental Guidance for Dermal Risk Assessment) (U.S. Environmental Protection Agency) (2004).

11.	Shi Z, Liu J, Song X, Wang Y, Li J, Wei S. The risk assessment and burden of cancer attributable to dietary cadmium exposure in China, 2019. *Environmental pollution (1987)*. 2025;368:125756. doi:10.1016/j.envpol.2025.125756

12.	Wang J, Gao P, Li M-Y, et al. Dermal bioaccessibility and cytotoxicity of heavy metals in urban soils from a typical plateau city: Implication for human health. *The Science of the total environment*. 2022;835:155544. doi:10.1016/j.scitotenv.2022.155544
